# 3-Benzyl-7-methyl-9-phenyl-2-tosyl-2,3,3a,4,9,9a-hexa­hydro-1*H*-pyrrolo[3,4-*b*]quinoline

**DOI:** 10.1107/S1600536809044481

**Published:** 2009-10-31

**Authors:** K. Chinnakali, D. Sudha, M. Jayagobi, R. Raghunathan, Hoong-Kun Fun

**Affiliations:** a Department of Physics, Anna University Chennai, Chennai 600 025, India; b Department of Organic Chemistry, University of Madras, Guindy Campus, Chennai 600 025, India; c X-ray Crystallography Unit, School of Physics, Universiti Sains Malaysia, 11800 USM, Penang, Malaysia

## Abstract

In the title compound, C_32_H_32_N_2_O_2_S, the pyrrolidine ring adopts a twist conformation while the tetra­hydro­pyridine ring is in a distorted half-chair conformation. The two rings are *trans*-fused. The dihedral angle between the sulfonyl and benzyl phenyl rings is 72.54 (14)°. The mol­ecular structure is stabilized by C—H⋯O hydrogen bonds, and N—H⋯π inter­actions involving the benzyl phenyl ring. The screw-related mol­ecules are linked into chains along the *b* axis by C—H⋯O hydrogen bonds and C—H⋯π inter­actions. Adjacent inversion-related chains inter­act *via* C—H⋯π inter­actions, forming a two-dimensional network parallel to the *bc* plane.

## Related literature

For the anti­cancer and photochemotherapeutic activity of pyrroloquinoline derivatives, see: Ferlin *et al.* (2005 ▶); Gasparotto *et al.* (2007 ▶); Barraja *et al.* (2003 ▶). For a related structure, see: Sudha *et al.* (2009 ▶). For ring puckering parameters, see: Cremer & Pople (1975 ▶). For asymmetry parameters, see: Duax *et al.* (1976 ▶).
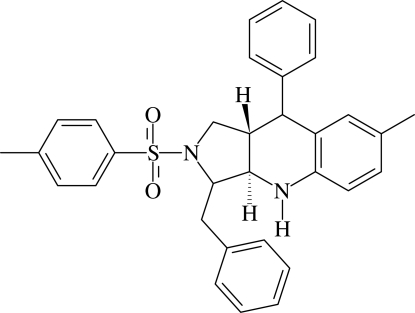


## Experimental

### 

#### Crystal data

C_32_H_32_N_2_O_2_S
*M* *_r_* = 508.66
Monoclinic, 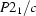

*a* = 9.0445 (4) Å
*b* = 10.6014 (4) Å
*c* = 27.533 (1) Å
β = 96.294 (3)°
*V* = 2624.07 (18) Å^3^
*Z* = 4
Mo *K*α radiation
μ = 0.16 mm^−1^
*T* = 100 K
0.38 × 0.20 × 0.17 mm

#### Data collection

Bruker SMART APEXII CCD area-detector diffractometer
Absorption correction: multi-scan (*SADABS*; Bruker, 2005 ▶) *T*_min_ = 0.360, *T*_max_ = 0.974
22823 measured reflections
5138 independent reflections
3615 reflections with *I* > 2σ(*I*)
*R*_int_ = 0.078

#### Refinement

*R*[*F* ^2^ > 2σ(*F* ^2^)] = 0.061
*wR*(*F* ^2^) = 0.178
*S* = 1.04
5138 reflections
340 parameters
H atoms treated by a mixture of independent and constrained refinement
Δρ_max_ = 0.69 e Å^−3^
Δρ_min_ = −0.51 e Å^−3^

### 

Data collection: *APEX2* (Bruker, 2005 ▶); cell refinement: *SAINT* (Bruker, 2005 ▶); data reduction: *SAINT*; program(s) used to solve structure: *SHELXTL* (Sheldrick, 2008 ▶); program(s) used to refine structure: *SHELXTL*; molecular graphics: *SHELXTL*; software used to prepare material for publication: *SHELXTL* and *PLATON* (Spek, 2009 ▶).

## Supplementary Material

Crystal structure: contains datablocks global, I. DOI: 10.1107/S1600536809044481/tk2561sup1.cif

Structure factors: contains datablocks I. DOI: 10.1107/S1600536809044481/tk2561Isup2.hkl

Additional supplementary materials:  crystallographic information; 3D view; checkCIF report

## Figures and Tables

**Table 1:** Hydrogen-bond geometry (Å, °)

*D*—H⋯*A*	*D*—H	H⋯*A*	*D*⋯*A*	*D*—H⋯*A*
C25—H25*A*⋯O2	0.97	2.56	3.182 (3)	122
C28—H28⋯O2^i^	0.93	2.49	3.282 (4)	143
N2—H1*N*2⋯*Cg*3	0.87 (3)	2.69 (3)	3.461 (3)	148 (2)
C3—H3⋯*Cg*3^i^	0.98	2.93	3.852 (3)	158
C18—H18*B*⋯*Cg*2^ii^	0.96	2.90	3.723 (4)	145
C21—H21⋯*Cg*1^iii^	0.93	2.74	3.637 (3)	162

Symmetry codes: (i) 
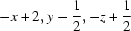
; (ii) 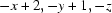; (iii) 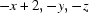. *Cg*1, *Cg*2 and *Cg*3 are the centroids of the C4–C9, C12–C17 and C26–C31 rings, respectively.

**Fig. 1.**
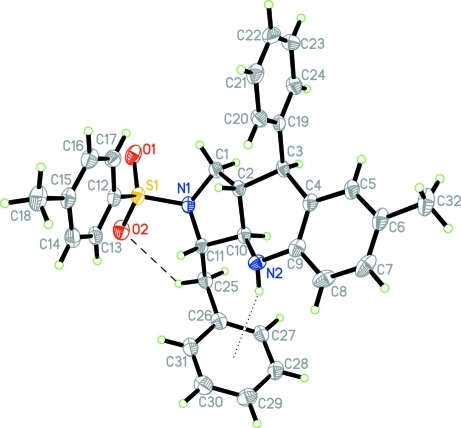
The molecular structure of the title compound. Displacement ellipsoids are drawn at the 50% probability level. The dashed line indicates a C—H···O hydrogen bond and the dotted line indicates an N—H···π interaction.

**Fig. 2.**
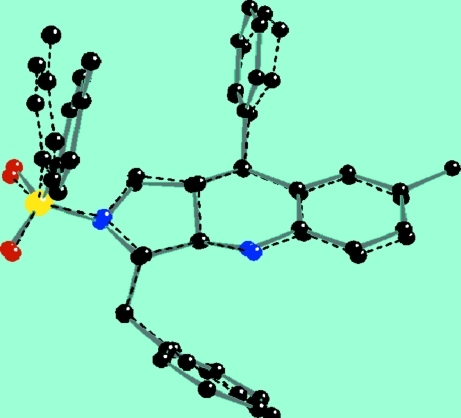
Fit of the title molecule (solid lines) with (II) (dashed lines). H atoms have been omitted for clarity.

**Fig. 3.**
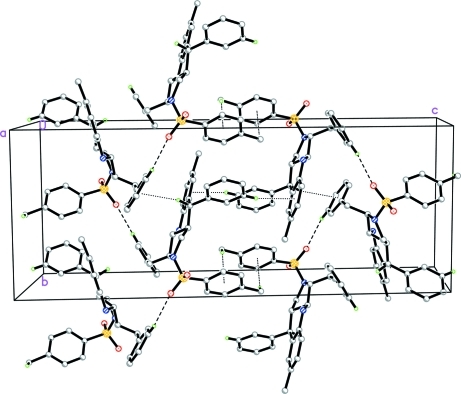
Crystal packing of the title compound. C—H···O hydrogen bonds are shown as dashed lines and C—H···π interactions are shown as dotted lines. For the sake of clarity, H atoms not involved in the interactions have been omitted.

## supplementary crystallographic information

### Comment

Pyrroloquinoline derivatives have been synthesized and investigated as potential anticancer drugs (Ferlin *et al.*, 2005; Gasparotto *et al.*, 2007). Some of them have been found to exhibit photochemotherapeutic activity (Barraja *et al.*, 2003). As part of our studies on pyrroloquinoline derivatives, we report here the crystal structure of the title compound (I).

In the title molecule, the pyrrolidine ring adopts a twist conformation; the asymmetry parameters ΔC_2_[C2—C10] (Duax *et al.*, 1976) and the puckering parameters q_2_ and φ (Cremer & Pople, 1975) are 6.1 (3)°, 0.407 (3) Å and 85.0 (4)°, respectively. The tosyl group is attached to the pyrrolidine ring in a biaxial position. The tetrahydropyridine ring adopts a distorted half-chair conformation; the Q, θ, φ and ΔC_s_[C10] values for the above ring are 0.522 (3) Å, 129.4 (3)°, 93.1 (3)° and 101.3 (4)°, respectively. The phenyl group attached to the tetrahydropyridine ring is in a biaxial position. The C19—C24 phenyl ring forms dihedral angles of 80.98 (13) and 7.40 (14)°, respectively, with the C4—C9 and C12—C17 benzene rings. The C12—C17 and C26—C31 rings are oriented at a dihedral angle of 72.54 (14)°. The molecular structure is stabilized by C—H···O hydrogen bonds and N—H···π interactions (Table 1).

Bond lengths and angles are comparable with those in 3-benzyl-9-phenyl-2-tosyl-2,3,3a,4,9,9a-hexahydro-1*H*-pyrrolo[3,4-*b*]quinoline, (II), (Sudha *et al.*, 2009). A superposition of the non-H atoms of the above molecule with those of the title molecule using *XP* in *SHELXTL* (Sheldrick, 2008), gave an r.m.s. deviation of 0.489 Å (Fig. 2). In both compounds, the pyrrolidine ring is *trans*-fused to the tetrahydropyridine ring but they differ in relative orientations of the phenyl rings.

In the solid state, screw-related molecules are linked into chains along the *b* axis by C—H···O hydrogen bonds and C—H···π interactions involving the C26—C31 ring (Table 1). Adjacent inversion-related chains interact *via* C—H···π interactions involving the C4—C9 and C12—C17 rings to form a two-dimensional network parallel to the *bc* plane (Fig.3).

A comparison of crystal packing in (I) and (II) shows that the presence of the methyl group at 7-position completely changes the packing mode. Without the methyl group, the molecules are linked into a chain along the *a* axis by intermolecular C—H···π interactions. But the presence of the methyl group resulted in a two-dimensional network parallel to the *bc* plane, as discussed above.

### Experimental

InCl_3_ (20 mol%) was added to a mixture of 2-(*N*-cinnamyl-*N*-tosylamino)-3-phenyl propanal (1 mmol) and *p*-methyl aniline (1 mmol) in acetonitrile (20 ml). The reaction mixture was stirred at room temperature for 1 min. On completion of the reaction, as indicated by TLC, the mixture was quenched with water and extracted with ethyl acetate. The organic layer was washed with brine and dried over Na_2_SO_4_. The solvent was evaporated *in vacuo* and the crude product was chromatographed on silica gel using a hexane-ethyl acetate (8.5:1.5 *v*/*v*) mixture to obtain the title compound. The compound was recrystallized from ethyl acetate solution by slow evaporation.

### Refinement

The N-bound H atom was located in a difference map and refined freely [N—H = 0.87 (3) Å]. The remaining H atoms were positioned geometrically (C—H = 0.93–0.98 Å) and allowed to ride on their parent atoms, with *U*_iso_(H) = 1.2*U*_eq_(C) or 1.5*U*_eq_(C_methyl_). A rotating group model was used for methyl groups. Reflection 002 was partially obscured by the beam stop and was omitted.

### Crystal data

**Table:**

C_32_H_32_N_2_O_2_S	*F*(000) = 1080
*M**_r_* = 508.66	*D*_x_ = 1.288 Mg m^−^^3^
Monoclinic, *P*2_1_/*c*	Mo *K*α radiation, λ = 0.71073 Å
Hall symbol: -P 2ybc	Cell parameters from 5605 reflections
*a* = 9.0445 (4) Å	θ = 2.3–30.1°
*b* = 10.6014 (4) Å	µ = 0.16 mm^−^^1^
*c* = 27.533 (1) Å	*T* = 100 K
β = 96.294 (3)°	Block, colourless
*V* = 2624.07 (18) Å^3^	0.38 × 0.20 × 0.17 mm
*Z* = 4	

### Data collection

**Table:**

Bruker SMART APEXII CCD area-detector diffractometer	5138 independent reflections
Radiation source: fine-focus sealed tube	3615 reflections with *I* > 2σ(*I*)
graphite	*R*_int_ = 0.078
φ and ω scans	θ_max_ = 26.0°, θ_min_ = 2.6°
Absorption correction: multi-scan (*SADABS*; Bruker, 2005)	*h* = −10→11
*T*_min_ = 0.360, *T*_max_ = 0.974	*k* = −13→12
22823 measured reflections	*l* = −33→33

### Refinement

**Table:**

Refinement on *F*^2^	Primary atom site location: structure-invariant direct methods
Least-squares matrix: full	Secondary atom site location: difference Fourier map
*R*[*F*^2^ > 2σ(*F*^2^)] = 0.061	Hydrogen site location: inferred from neighbouring sites
*wR*(*F*^2^) = 0.178	H atoms treated by a mixture of independent and constrained refinement
*S* = 1.04	*w* = 1/[σ^2^(*F*_o_^2^) + (0.1011*P*)^2^ + 1.1335*P*] where *P* = (*F*_o_^2^ + 2*F*_c_^2^)/3
5138 reflections	(Δ/σ)_max_ = 0.001
340 parameters	Δρ_max_ = 0.69 e Å^−^^3^
0 restraints	Δρ_min_ = −0.50 e Å^−^^3^

### Special details

**Table:**

Geometry. All e.s.d.'s (except the e.s.d. in the dihedral angle between two l.s. planes) are estimated using the full covariance matrix. The cell e.s.d.'s are taken into account individually in the estimation of e.s.d.'s in distances, angles and torsion angles; correlations between e.s.d.'s in cell parameters are only used when they are defined by crystal symmetry. An approximate (isotropic) treatment of cell e.s.d.'s is used for estimating e.s.d.'s involving l.s. planes.
Refinement. Refinement of *F*^2^ against ALL reflections. The weighted *R*-factor *wR* and goodness of fit *S* are based on *F*^2^, conventional *R*-factors *R* are based on *F*, with *F* set to zero for negative *F*^2^. The threshold expression of *F*^2^ > σ(*F*^2^) is used only for calculating *R*-factors(gt) *etc*. and is not relevant to the choice of reflections for refinement. *R*-factors based on *F*^2^ are statistically about twice as large as those based on *F*, and *R*- factors based on ALL data will be even larger.

### Fractional atomic coordinates and isotropic or equivalent isotropic displacement parameters (Å^2^)

**Table:**

	*x*	*y*	*z*	*U*_iso_*/*U*_eq_	
S1	0.62265 (8)	0.48132 (7)	0.13646 (2)	0.0262 (2)	
O1	0.4733 (2)	0.4422 (2)	0.12298 (7)	0.0322 (5)	
O2	0.6528 (2)	0.58710 (19)	0.16844 (7)	0.0335 (5)	
N1	0.7118 (3)	0.3614 (2)	0.16335 (8)	0.0239 (5)	
N2	1.0859 (3)	0.2372 (2)	0.15932 (8)	0.0249 (5)	
H1N2	1.156 (3)	0.272 (3)	0.1791 (11)	0.022 (7)*	
C1	0.6810 (3)	0.2332 (3)	0.14178 (10)	0.0260 (6)	
H1A	0.6104	0.2378	0.1126	0.031*	
H1B	0.6422	0.1770	0.1651	0.031*	
C2	0.8318 (3)	0.1896 (2)	0.12960 (8)	0.0214 (6)	
H2	0.8528	0.2304	0.0992	0.026*	
C3	0.8577 (3)	0.0474 (2)	0.12508 (9)	0.0217 (6)	
H3	0.8140	0.0064	0.1520	0.026*	
C4	1.0250 (3)	0.0211 (3)	0.13221 (8)	0.0228 (6)	
C5	1.0783 (3)	−0.0993 (3)	0.12259 (9)	0.0264 (6)	
H5	1.0096	−0.1616	0.1122	0.032*	
C6	1.2275 (3)	−0.1305 (3)	0.12773 (9)	0.0305 (7)	
C7	1.3287 (4)	−0.0369 (3)	0.14395 (10)	0.0362 (8)	
H7	1.4300	−0.0551	0.1478	0.043*	
C8	1.2805 (3)	0.0830 (3)	0.15445 (10)	0.0333 (7)	
H8	1.3500	0.1441	0.1655	0.040*	
C9	1.1304 (3)	0.1136 (3)	0.14877 (9)	0.0258 (6)	
C10	0.9342 (3)	0.2446 (2)	0.17126 (9)	0.0223 (6)	
H10	0.9251	0.1957	0.2010	0.027*	
C11	0.8745 (3)	0.3775 (2)	0.17768 (9)	0.0214 (6)	
H11	0.9149	0.4339	0.1542	0.026*	
C12	0.7040 (3)	0.5160 (3)	0.08227 (9)	0.0262 (6)	
C13	0.8142 (3)	0.6074 (3)	0.08287 (10)	0.0296 (7)	
H13	0.8492	0.6472	0.1120	0.036*	
C14	0.8717 (4)	0.6392 (3)	0.03979 (10)	0.0332 (7)	
H14	0.9459	0.7000	0.0404	0.040*	
C15	0.8201 (3)	0.5815 (3)	−0.00429 (10)	0.0293 (7)	
C16	0.7127 (3)	0.4883 (3)	−0.00385 (10)	0.0307 (7)	
H16	0.6792	0.4474	−0.0329	0.037*	
C17	0.6536 (3)	0.4543 (3)	0.03907 (10)	0.0290 (6)	
H17	0.5817	0.3914	0.0388	0.035*	
C18	0.8756 (4)	0.6232 (3)	−0.05135 (11)	0.0381 (8)	
H18A	0.8361	0.5685	−0.0774	0.057*	
H18B	0.9823	0.6194	−0.0481	0.057*	
H18C	0.8439	0.7082	−0.0586	0.057*	
C19	0.7819 (3)	−0.0079 (2)	0.07772 (9)	0.0219 (6)	
C20	0.8280 (3)	0.0268 (3)	0.03263 (9)	0.0270 (6)	
H20	0.9051	0.0844	0.0316	0.032*	
C21	0.7597 (3)	−0.0241 (3)	−0.01055 (10)	0.0305 (7)	
H21	0.7916	−0.0010	−0.0403	0.037*	
C22	0.6438 (3)	−0.1093 (3)	−0.00949 (11)	0.0327 (7)	
H22	0.5974	−0.1428	−0.0385	0.039*	
C23	0.5976 (3)	−0.1444 (3)	0.03477 (11)	0.0329 (7)	
H23	0.5202	−0.2018	0.0357	0.040*	
C24	0.6674 (3)	−0.0936 (3)	0.07798 (10)	0.0276 (6)	
H24	0.6360	−0.1180	0.1076	0.033*	
C25	0.9111 (3)	0.4321 (3)	0.22950 (9)	0.0261 (6)	
H25A	0.8648	0.5142	0.2315	0.031*	
H25B	0.8722	0.3769	0.2532	0.031*	
C26	1.0768 (3)	0.4441 (3)	0.24082 (9)	0.0257 (6)	
C27	1.1623 (3)	0.3525 (3)	0.26768 (9)	0.0263 (6)	
H27	1.1146	0.2873	0.2823	0.032*	
C28	1.3159 (4)	0.3569 (3)	0.27287 (9)	0.0307 (7)	
H28	1.3702	0.2939	0.2903	0.037*	
C29	1.3898 (3)	0.4552 (3)	0.25217 (10)	0.0332 (7)	
H29	1.4932	0.4583	0.2556	0.040*	
C30	1.3068 (4)	0.5486 (3)	0.22638 (10)	0.0342 (7)	
H30	1.3549	0.6153	0.2128	0.041*	
C31	1.1532 (3)	0.5429 (3)	0.22085 (9)	0.0293 (7)	
H31	1.0993	0.6062	0.2035	0.035*	
C32	1.2786 (4)	−0.2591 (3)	0.11267 (11)	0.0403 (8)	
H32A	1.2094	−0.3220	0.1210	0.060*	
H32B	1.3752	−0.2767	0.1294	0.060*	
H32C	1.2837	−0.2603	0.0780	0.060*	

### Atomic displacement parameters (Å^2^)

**Table:**

	*U*^11^	*U*^22^	*U*^33^	*U*^12^	*U*^13^	*U*^23^
S1	0.0340 (4)	0.0233 (4)	0.0212 (3)	0.0093 (3)	0.0028 (3)	−0.0022 (3)
O1	0.0324 (12)	0.0364 (12)	0.0282 (10)	0.0140 (9)	0.0044 (8)	0.0006 (9)
O2	0.0486 (13)	0.0266 (11)	0.0254 (10)	0.0114 (9)	0.0040 (9)	−0.0076 (8)
N1	0.0294 (13)	0.0202 (12)	0.0219 (11)	0.0044 (9)	0.0023 (9)	0.0017 (9)
N2	0.0245 (13)	0.0277 (13)	0.0221 (11)	0.0020 (10)	0.0007 (10)	−0.0082 (10)
C1	0.0295 (16)	0.0213 (15)	0.0268 (14)	0.0015 (12)	0.0015 (11)	−0.0008 (11)
C2	0.0293 (15)	0.0188 (14)	0.0161 (12)	0.0011 (11)	0.0033 (11)	0.0033 (10)
C3	0.0303 (16)	0.0183 (14)	0.0170 (12)	0.0010 (11)	0.0054 (10)	0.0038 (10)
C4	0.0305 (16)	0.0250 (15)	0.0127 (11)	0.0046 (12)	0.0018 (10)	0.0019 (10)
C5	0.0384 (17)	0.0252 (15)	0.0153 (12)	0.0051 (12)	0.0015 (11)	0.0047 (10)
C6	0.0436 (19)	0.0342 (17)	0.0134 (12)	0.0147 (14)	0.0012 (12)	0.0039 (11)
C7	0.0331 (18)	0.054 (2)	0.0205 (13)	0.0201 (15)	−0.0019 (12)	−0.0069 (13)
C8	0.0294 (17)	0.048 (2)	0.0214 (13)	0.0040 (14)	−0.0010 (12)	−0.0121 (13)
C9	0.0318 (16)	0.0328 (16)	0.0125 (11)	0.0063 (12)	0.0013 (11)	−0.0017 (11)
C10	0.0297 (16)	0.0212 (14)	0.0163 (12)	0.0029 (11)	0.0035 (10)	0.0008 (10)
C11	0.0271 (15)	0.0193 (14)	0.0177 (12)	0.0027 (11)	0.0015 (10)	0.0010 (10)
C12	0.0345 (17)	0.0234 (15)	0.0206 (12)	0.0089 (12)	0.0023 (11)	0.0016 (11)
C13	0.0454 (19)	0.0191 (14)	0.0231 (13)	0.0035 (13)	−0.0016 (12)	0.0006 (11)
C14	0.0408 (19)	0.0254 (16)	0.0326 (15)	0.0012 (13)	0.0004 (13)	0.0060 (12)
C15	0.0352 (17)	0.0258 (16)	0.0269 (14)	0.0114 (13)	0.0029 (12)	0.0032 (11)
C16	0.0402 (18)	0.0306 (16)	0.0206 (13)	0.0107 (13)	−0.0001 (12)	−0.0049 (11)
C17	0.0324 (17)	0.0258 (16)	0.0281 (14)	0.0046 (12)	−0.0006 (12)	−0.0029 (11)
C18	0.048 (2)	0.0374 (18)	0.0297 (15)	0.0112 (15)	0.0075 (14)	0.0067 (13)
C19	0.0264 (15)	0.0167 (13)	0.0223 (12)	0.0039 (11)	0.0013 (11)	0.0025 (10)
C20	0.0370 (17)	0.0226 (15)	0.0212 (13)	−0.0017 (12)	0.0022 (11)	0.0005 (11)
C21	0.0390 (18)	0.0312 (16)	0.0207 (13)	0.0076 (13)	0.0009 (12)	0.0016 (12)
C22	0.0355 (18)	0.0304 (16)	0.0295 (14)	0.0064 (13)	−0.0091 (12)	−0.0080 (12)
C23	0.0290 (17)	0.0281 (17)	0.0401 (16)	−0.0035 (12)	−0.0034 (13)	−0.0031 (13)
C24	0.0324 (17)	0.0234 (15)	0.0275 (14)	0.0017 (12)	0.0053 (12)	−0.0008 (11)
C25	0.0360 (17)	0.0225 (14)	0.0201 (13)	0.0025 (12)	0.0045 (11)	−0.0036 (11)
C26	0.0376 (17)	0.0241 (15)	0.0159 (12)	0.0001 (12)	0.0048 (11)	−0.0054 (10)
C27	0.0417 (18)	0.0212 (14)	0.0152 (12)	−0.0024 (12)	−0.0006 (11)	−0.0024 (10)
C28	0.0417 (19)	0.0287 (16)	0.0203 (13)	0.0059 (13)	−0.0035 (12)	−0.0023 (11)
C29	0.0324 (17)	0.0429 (19)	0.0236 (14)	−0.0015 (14)	−0.0002 (12)	−0.0016 (13)
C30	0.046 (2)	0.0344 (18)	0.0225 (13)	−0.0070 (14)	0.0031 (13)	0.0022 (12)
C31	0.0433 (19)	0.0231 (15)	0.0208 (13)	0.0022 (13)	0.0006 (12)	−0.0008 (11)
C32	0.053 (2)	0.0387 (19)	0.0301 (15)	0.0231 (15)	0.0078 (14)	0.0043 (13)

### Geometric parameters (Å, °)

**Table:**

S1—O1	1.423 (2)	C14—H14	0.93
S1—O2	1.433 (2)	C15—C16	1.387 (4)
S1—N1	1.638 (2)	C15—C18	1.506 (4)
S1—C12	1.773 (3)	C16—C17	1.397 (4)
N1—C11	1.491 (3)	C16—H16	0.93
N1—C1	1.497 (3)	C17—H17	0.93
N2—C9	1.410 (4)	C18—H18A	0.96
N2—C10	1.448 (3)	C18—H18B	0.96
N2—H1N2	0.87 (3)	C18—H18C	0.96
C1—C2	1.512 (4)	C19—C24	1.379 (4)
C1—H1A	0.97	C19—C20	1.401 (4)
C1—H1B	0.97	C20—C21	1.388 (4)
C2—C10	1.510 (4)	C20—H20	0.93
C2—C3	1.532 (4)	C21—C22	1.386 (4)
C2—H2	0.98	C21—H21	0.93
C3—C19	1.522 (4)	C22—C23	1.382 (4)
C3—C4	1.530 (4)	C22—H22	0.93
C3—H3	0.98	C23—C24	1.392 (4)
C4—C5	1.400 (4)	C23—H23	0.93
C4—C9	1.408 (4)	C24—H24	0.93
C5—C6	1.382 (4)	C25—C26	1.502 (4)
C5—H5	0.93	C25—H25A	0.97
C6—C7	1.391 (5)	C25—H25B	0.97
C6—C32	1.512 (4)	C26—C31	1.400 (4)
C7—C8	1.385 (4)	C26—C27	1.401 (4)
C7—H7	0.93	C27—C28	1.381 (4)
C8—C9	1.388 (4)	C27—H27	0.93
C8—H8	0.93	C28—C29	1.394 (4)
C10—C11	1.527 (4)	C28—H28	0.93
C10—H10	0.98	C29—C30	1.390 (4)
C11—C25	1.541 (3)	C29—H29	0.93
C11—H11	0.98	C30—C31	1.383 (4)
C12—C13	1.389 (4)	C30—H30	0.93
C12—C17	1.390 (4)	C31—H31	0.93
C13—C14	1.388 (4)	C32—H32A	0.96
C13—H13	0.93	C32—H32B	0.96
C14—C15	1.393 (4)	C32—H32C	0.96
			
O1—S1—O2	119.99 (12)	C13—C14—C15	121.1 (3)
O1—S1—N1	107.33 (12)	C13—C14—H14	119.4
O2—S1—N1	106.16 (12)	C15—C14—H14	119.4
O1—S1—C12	108.10 (13)	C16—C15—C14	118.2 (3)
O2—S1—C12	106.62 (13)	C16—C15—C18	121.1 (3)
N1—S1—C12	108.18 (12)	C14—C15—C18	120.6 (3)
C11—N1—C1	110.2 (2)	C15—C16—C17	121.7 (3)
C11—N1—S1	116.96 (17)	C15—C16—H16	119.2
C1—N1—S1	117.64 (17)	C17—C16—H16	119.2
C9—N2—C10	113.3 (2)	C12—C17—C16	118.8 (3)
C9—N2—H1N2	108.1 (19)	C12—C17—H17	120.6
C10—N2—H1N2	118.8 (19)	C16—C17—H17	120.6
N1—C1—C2	103.4 (2)	C15—C18—H18A	109.5
N1—C1—H1A	111.1	C15—C18—H18B	109.5
C2—C1—H1A	111.1	H18A—C18—H18B	109.5
N1—C1—H1B	111.1	C15—C18—H18C	109.5
C2—C1—H1B	111.1	H18A—C18—H18C	109.5
H1A—C1—H1B	109.0	H18B—C18—H18C	109.5
C10—C2—C1	101.9 (2)	C24—C19—C20	118.3 (2)
C10—C2—C3	110.8 (2)	C24—C19—C3	121.1 (2)
C1—C2—C3	117.9 (2)	C20—C19—C3	120.6 (2)
C10—C2—H2	108.6	C21—C20—C19	120.6 (3)
C1—C2—H2	108.6	C21—C20—H20	119.7
C3—C2—H2	108.6	C19—C20—H20	119.7
C19—C3—C4	112.7 (2)	C22—C21—C20	120.1 (3)
C19—C3—C2	113.0 (2)	C22—C21—H21	119.9
C4—C3—C2	109.1 (2)	C20—C21—H21	119.9
C19—C3—H3	107.3	C23—C22—C21	119.8 (3)
C4—C3—H3	107.3	C23—C22—H22	120.1
C2—C3—H3	107.3	C21—C22—H22	120.1
C5—C4—C9	117.5 (3)	C22—C23—C24	119.8 (3)
C5—C4—C3	119.9 (2)	C22—C23—H23	120.1
C9—C4—C3	122.6 (2)	C24—C23—H23	120.1
C6—C5—C4	123.5 (3)	C19—C24—C23	121.4 (3)
C6—C5—H5	118.3	C19—C24—H24	119.3
C4—C5—H5	118.3	C23—C24—H24	119.3
C5—C6—C7	117.6 (3)	C26—C25—C11	109.4 (2)
C5—C6—C32	120.8 (3)	C26—C25—H25A	109.8
C7—C6—C32	121.4 (3)	C11—C25—H25A	109.8
C8—C7—C6	120.7 (3)	C26—C25—H25B	109.8
C8—C7—H7	119.6	C11—C25—H25B	109.8
C6—C7—H7	119.6	H25A—C25—H25B	108.2
C7—C8—C9	121.2 (3)	C31—C26—C27	117.3 (3)
C7—C8—H8	119.4	C31—C26—C25	120.4 (2)
C9—C8—H8	119.4	C27—C26—C25	122.1 (3)
C8—C9—C4	119.5 (3)	C28—C27—C26	121.5 (3)
C8—C9—N2	119.5 (3)	C28—C27—H27	119.2
C4—C9—N2	121.0 (3)	C26—C27—H27	119.2
N2—C10—C2	108.9 (2)	C27—C28—C29	120.3 (3)
N2—C10—C11	115.6 (2)	C27—C28—H28	119.9
C2—C10—C11	104.4 (2)	C29—C28—H28	119.9
N2—C10—H10	109.2	C30—C29—C28	119.1 (3)
C2—C10—H10	109.2	C30—C29—H29	120.5
C11—C10—H10	109.2	C28—C29—H29	120.5
N1—C11—C10	102.4 (2)	C31—C30—C29	120.3 (3)
N1—C11—C25	113.2 (2)	C31—C30—H30	119.8
C10—C11—C25	114.3 (2)	C29—C30—H30	119.8
N1—C11—H11	108.9	C30—C31—C26	121.5 (3)
C10—C11—H11	108.9	C30—C31—H31	119.2
C25—C11—H11	108.9	C26—C31—H31	119.2
C13—C12—C17	120.4 (2)	C6—C32—H32A	109.5
C13—C12—S1	119.9 (2)	C6—C32—H32B	109.5
C17—C12—S1	119.7 (2)	H32A—C32—H32B	109.5
C14—C13—C12	119.7 (3)	C6—C32—H32C	109.5
C14—C13—H13	120.2	H32A—C32—H32C	109.5
C12—C13—H13	120.2	H32B—C32—H32C	109.5
			
O1—S1—N1—C11	175.82 (17)	C2—C10—C11—N1	31.8 (2)
O2—S1—N1—C11	−54.7 (2)	N2—C10—C11—C25	−85.8 (3)
C12—S1—N1—C11	59.4 (2)	C2—C10—C11—C25	154.5 (2)
O1—S1—N1—C1	41.1 (2)	O1—S1—C12—C13	149.6 (2)
O2—S1—N1—C1	170.60 (19)	O2—S1—C12—C13	19.4 (3)
C12—S1—N1—C1	−75.3 (2)	N1—S1—C12—C13	−94.4 (2)
C11—N1—C1—C2	−16.8 (3)	O1—S1—C12—C17	−28.3 (3)
S1—N1—C1—C2	120.77 (19)	O2—S1—C12—C17	−158.5 (2)
N1—C1—C2—C10	35.8 (2)	N1—S1—C12—C17	87.6 (2)
N1—C1—C2—C3	157.3 (2)	C17—C12—C13—C14	1.5 (4)
C10—C2—C3—C19	−167.9 (2)	S1—C12—C13—C14	−176.5 (2)
C1—C2—C3—C19	75.3 (3)	C12—C13—C14—C15	0.4 (4)
C10—C2—C3—C4	−41.8 (3)	C13—C14—C15—C16	−2.0 (4)
C1—C2—C3—C4	−158.6 (2)	C13—C14—C15—C18	175.7 (3)
C19—C3—C4—C5	−44.2 (3)	C14—C15—C16—C17	1.7 (4)
C2—C3—C4—C5	−170.5 (2)	C18—C15—C16—C17	−175.9 (3)
C19—C3—C4—C9	136.4 (2)	C13—C12—C17—C16	−1.7 (4)
C2—C3—C4—C9	10.1 (3)	S1—C12—C17—C16	176.2 (2)
C9—C4—C5—C6	−1.1 (4)	C15—C16—C17—C12	0.1 (4)
C3—C4—C5—C6	179.5 (2)	C4—C3—C19—C24	121.5 (3)
C4—C5—C6—C7	0.9 (4)	C2—C3—C19—C24	−114.3 (3)
C4—C5—C6—C32	−174.8 (2)	C4—C3—C19—C20	−58.1 (3)
C5—C6—C7—C8	0.0 (4)	C2—C3—C19—C20	66.1 (3)
C32—C6—C7—C8	175.6 (3)	C24—C19—C20—C21	0.0 (4)
C6—C7—C8—C9	−0.5 (4)	C3—C19—C20—C21	179.6 (2)
C7—C8—C9—C4	0.2 (4)	C19—C20—C21—C22	0.5 (4)
C7—C8—C9—N2	−179.3 (3)	C20—C21—C22—C23	−0.6 (4)
C5—C4—C9—C8	0.5 (3)	C21—C22—C23—C24	0.2 (5)
C3—C4—C9—C8	179.9 (2)	C20—C19—C24—C23	−0.4 (4)
C5—C4—C9—N2	−179.9 (2)	C3—C19—C24—C23	179.9 (3)
C3—C4—C9—N2	−0.6 (4)	C22—C23—C24—C19	0.3 (4)
C10—N2—C9—C8	−156.3 (2)	N1—C11—C25—C26	179.6 (2)
C10—N2—C9—C4	24.2 (3)	C10—C11—C25—C26	62.9 (3)
C9—N2—C10—C2	−56.6 (3)	C11—C25—C26—C31	76.8 (3)
C9—N2—C10—C11	−173.7 (2)	C11—C25—C26—C27	−97.3 (3)
C1—C2—C10—N2	−166.6 (2)	C31—C26—C27—C28	−2.2 (4)
C3—C2—C10—N2	67.1 (3)	C25—C26—C27—C28	172.1 (2)
C1—C2—C10—C11	−42.5 (2)	C26—C27—C28—C29	1.5 (4)
C3—C2—C10—C11	−168.8 (2)	C27—C28—C29—C30	0.1 (4)
C1—N1—C11—C10	−9.1 (2)	C28—C29—C30—C31	−0.8 (4)
S1—N1—C11—C10	−146.95 (17)	C29—C30—C31—C26	0.0 (4)
C1—N1—C11—C25	−132.6 (2)	C27—C26—C31—C30	1.5 (4)
S1—N1—C11—C25	89.6 (2)	C25—C26—C31—C30	−173.0 (2)
N2—C10—C11—N1	151.4 (2)		

### Hydrogen-bond geometry (Å, °)

**Table:**

*D*—H···*A*	*D*—H	H···*A*	*D*···*A*	*D*—H···*A*
C1—H1A···O1	0.97	2.53	2.914 (4)	104
C13—H13···O2	0.93	2.57	2.912 (3)	103
C25—H25A···O2	0.97	2.56	3.182 (3)	122
C28—H28···O2^i^	0.93	2.49	3.282 (4)	143
N2—H1N2···Cg3	0.87 (3)	2.69 (3)	3.461 (3)	148 (2)
C3—H3···Cg3^i^	0.98	2.93	3.852 (3)	158
C18—H18B···Cg2^ii^	0.96	2.90	3.723 (4)	145
C21—H21···Cg1^iii^	0.93	2.74	3.637 (3)	162

Symmetry codes: (i) −*x*+2, *y*−1/2, −*z*+1/2; (ii) −*x*+2, −*y*+1, −*z*; (iii) −*x*+2, −*y*, −*z*.
